# *Bacillus* sp. FPF-1 Produced Keratinase with High Potential for Chicken Feather Degradation

**DOI:** 10.3390/molecules25071505

**Published:** 2020-03-26

**Authors:** Nonso E. Nnolim, Anthony I. Okoh, Uchechukwu U. Nwodo

**Affiliations:** 1 SA-MRC Microbial Water Quality Monitoring Centre, University of Fort Hare, Alice, 5700 Eastern Cape, South Africa; AOkoh@ufh.ac.za (A.I.O.); UNwodo@ufh.ac.za (U.U.N.); 2 Applied and Environmental Microbiology Research Group (AEMREG), Department of Biochemistry and Microbiology, University of Fort Hare, Private Bag X1314, Alice, 5700 Eastern Cape, South Africa

**Keywords:** biodegradation, chicken feathers, keratinase, valorization

## Abstract

Chicken feathers are predominantly composed of keratin; hence, valorizing the wastes becomes an imperative. In view of this, we isolated keratinase-producing bacteria and identified them through the 16S rDNA sequence. The process condition for keratinase activity was optimized, and electron micrography of the degradation timelines was determined. Keratinolytic bacteria were isolated and identified as *Bacillus* sp. FPF-1, *Chryseobacterium* sp. FPF-8, *Brevibacillus* sp. Nnolim-K2, *Brevibacillus* sp. FPF-12 and *Brevibacillus* sp. FSS-1; and their respective nucleotide sequences were deposited in GenBank, with the accession numbers MG214993, MG214994, MG214995, MG214996 and MG214999. The degree of feather degradation and keratinase concentration among the isolates ranged from 62.5 ± 2.12 to 86.0 ± 1.41(%) and 214.55 ± 5.14 to 440.01 ± 20.57 (U/mL), respectively. In the same vein, 0.1% (*w*/*v*) xylose, 0.5% (*w*/*v*) chicken feather, an initial fermentation pH of 5.0, fermentation temperature of 25 °C and an agitation speed of 150 rpm, respectively, served as the optimal physicochemical conditions for keratinase activity by *Bacillus* sp. FPF-1. The time course showed that *Bacillus* sp. FPF-1 yielded a keratinase concentration of 1698.18 ± 53.99(U/mL) at 120 h. The electron microscopic imaging showed completely structural dismemberment of intact chicken feather. *Bacillus* sp. FPF-1 holds great potential in the valorization of recalcitrant keratinous biomass from the agro sector into useful products.

## 1. Introduction

Chicken feathers are a significant waste product from poultry processing farms, and the disposal process has remained incineration, commuting to landfills and limited use as non-nutritive additives of feed. The composition of chicken feathers includes keratin, which bestows the mechanical stability associated with chicken feathers, leading to the recalcitrance to degradation [1]. The estimated abundance of crude protein in the avian feather has been approximated to about 82.36% [2], and this significant protein content portends an excellent opportunity for the valorization of the agro-waste biomass to high-value, cost-effective and renewable-source proteins for various applications, including livestock-feed supplementation. Notwithstanding the potentials presented by avian feathers as a high-protein source, the ability to dismember the feathers into functional components becomes the key for the valorization of the agro-waste. The traditional approach for the valorization of feathers involves endergonic reactions and chemical treatments [3]; these methods are capital intensive, and they produce a poorly digestible protein which is unsatisfactory as a feed supplement. Besides, the application of the methods also incorporates other compounds with no known biological importance [4]. 

Conversely, the use of the microbial-based technique for the dismemberment of the complex structures constituting the avian feather for high-value products has gained considerable traction. The bio-based approach has been adjudged as eco-friendly, energy-conserving and the protein yields are of high quality [5]. Consequently, the biodegradation of feather, therefore, represents a cost-effective and renewable approach for the valorization of agro-waste to high-value products. 

An account of bacterial and fungal species showing the potentials for the degradation of avian feathers abound. However, most of the microbes with excellent keratinolytic properties have been reported to exist among the dermatophytes, and this creates limitations for useful application [5,6]. Keratinolytic bacteria, on the other hand, present a litany of advantage over the fungal counterpart, including a high rate of extracellular proteolytic enzyme production [7]. The bio-catalytic efficiency of the keratinases produced by bacteria species tends toward a broad spectrum of proteinaceous substrates and robust at extreme conditions [8].

Considering the promising applicability of keratinase in green technology, it became imperative to explore some environment for bacterial diversity with the potentials to produce novel keratinases [6]. Nonetheless, the footprint of keratinases in the market is relatively low, and this further indicates that an opportunity abounds in the endeavor of obtaining keratinases with novel function. Therefore, this study was undertaken to assess the keratinolytic potentials of some bacteria isolated from poultry-composting sites and to optimize the process conditions for keratinolytic activity.

## 2. Results and Discussion

### 2.1. Identification of the Keratinase-Producing Bacteria

The 16S rDNA nucleotide sequences BLAST showed the isolate coded as FPF-1 to have a high sequence homology (100%) with *Bacillus cereus* AB1 (MF800922) and *Bacillus thuringiensis* WG20 (KY085971); hence, the isolate was identified as *Bacillus* sp. FPF-1, with an accession number MG214993. The other isolates coded as FPF-10, FPF-12 and FSS-1 showed 99% sequence homology with *Brevibacillus parabrevis* C20 (KX832699), *Brevibacillus parabrevis* NAP3 (KJ872854) and *Brevibacillus* sp. BAB-6437 (KY672924), respectively. Consequently, they were identified as *Brevibacillus* sp. Nnolim-K2, *Brevibacillus* sp. FPF-12 and *Brevibacillus* sp. FSS-1. The nucleotide sequences of the isolates were deposited in GenBank, with the respective accession numbers MG214995, MG214996 and MG214999, as shown in Table 1. Lastly, the isolate coded as FPF-8 showed 98% sequence homology with *Chryseobacterium culicis* R4-1A (NR_117008); therefore, it was identified as *Chryseobacterium* sp. FPF-8, and the nucleotide sequence was deposited in GenBank, with accession number MG214994. 

### 2.2. Evaluation for Keratinolytic Activity

**Figure 1:**
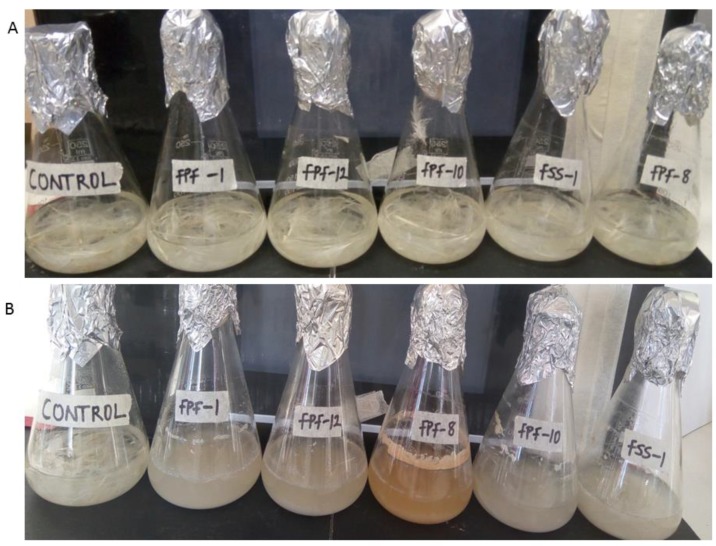
Biodegradation of intact chicken feathers by the keratinolytic bacterial isolates: (**A**) before incubation; and (**B**) after 96 h of incubation.

The hydrolysis of casein in skimmed milk (halo zones formation on skimmed milk agar) was an indication that the isolates produced extracellular proteases. Protease activity is relevant and a significant indication of the presences of keratinases. The screening for protease activity as a potential indicator for keratinase activity is a standard approach for screening and selection of potential keratinolytic bacteria [9]. The isolates coded as FPF-1, FPF-8, FPF-10, FPF-12 and FSS-1 showed significant feather degradation in basal medium with chicken feathers as a sole source of carbon and nitrogen (Figure 1). Consequently, these isolates were used in subsequent studies. The flask containing isolate coded FPF-8 showed orange-colored broth, which is characteristic of flexirubin-producing *Chryseobacterium* species [10].

The residual feathers were harvested and quantified by using the formula in Equation (1), at the end of fermentation duration, and the degree of degradation was 86.0 ± 1.41 (%) against *Bacillus* sp. FPF-1, 82.0 ± 1.41 (%) against *Chryseobacterium* sp. FPF-8, 66.0 ± 2.83 (%) against *Brevibacillus* sp. Nnolim-K2, 62.5 ± 2.12 (%) against *Brevibacillus* sp. FPF-12 and 69.5 ± 2.12 (%) against *Brevibacillus* sp. FSS-1 (Table 2). The pH of the cell-free broth, the concentrations of keratinase, total protein and thiol of the same cell-free broth were analyzed. The extracellular keratinase activity by the isolates ranged from 214.55 ± 5.14 (U/mL) against *Brevibacillus* sp. Nnolim-K2 to 440.01 ± 20.57 (U/mL) against *Bacillus* sp. FPF-1. Perhaps it would be prudent to indicate, at this point, that keratinases mediated the degradation of chicken feathers, as ample quantities of the enzymes were present in the cell-free broth. The bacterial isolates thrived on the keratinous biomass as the sole carbon and nitrogen source. In no small degree of certainty, it would be safe to indicate that the isolates produced keratinases [11]. Keratin-degrading efficiency of the test bacteria may be a reflection of the effectiveness of the extracellular keratinase produced; and the bacterial isolates under investigation showed consistency in that regard [12]. 

The concentration of thiols in the fermentation broth ranged from 461.69 ± 5.53 (μM) against *Brevibacillus* sp. FPF-12 to 2206.59 ± 49.79 (μM) against *Bacillus* sp. FPF-1. Disulfide bond reduction was identified to promote complete decomposition of keratin through structural modification significantly; and such structural alteration increases the vulnerability of keratin to proteolytic hydrolysis [13]. The pH of the fermented broth drifted from slightly acidic (initial fermentation pH) to alkaline; 7.35 ± 0.00 against *Brevibacillus* sp. FPF-12 to 8.53 ± 0.02 against *Chryseobacterium* sp. FPF-8 (Table 2). Biodegradation of keratinous biomass has been reported to instigate ammonification of the fermentation medium [14], which causes a drift in the medium pH toward the alkaline spectrum.

Consequently, the process of alkalization of the medium causes substrate swelling, which ultimately weakens the mechanical stability of keratin, hence, yielding to more efficient keratinolytic attack [15]—considering the keratinolytic activities of the bacterial isolates, *Bacillus* sp. FPF-1 displayed unusual keratinolytic activity; therefore, it was selected for the rest of the studies reported afterward.

### 2.3. Optimization of Extracellular Keratinase Activity and Total Protein Production by Bacillus sp. FPF-1

Upon evaluating the influence of initial fermentation pH on keratinase activity, respectively, fermentation occurred at pH 4.0 to 10.0, and the observation was that *Bacillus* sp. FPF-1 produced keratinase at all the pH for which fermentation was initiated. However, *Bacillus* sp. FPF-1 optimally produced keratinase at weak acidic pH 5.0.

**Figure 2:**
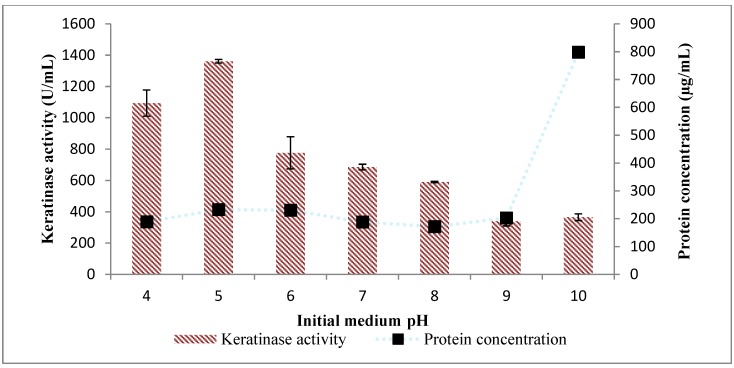
Effect of initial cultivation pH on keratinase activity by *Bacillus* sp. FPF-1.

Conversely, the total protein concentration in the broth increased as the pH tended toward alkaline and was markedly high with the initial formation pH 10.0 (Figure 2). The reason behind the observed phenomenon is not apparent. However, a logical interpretation may be that the test bacteria produced other enzymes, which cleaved the chicken feather into protein subunits, different from keratinases. A few studies have reported an initial ambient pH from weak acidic to neutral [16,17], and this observation is in accord with our findings.

With *Bacillus* species in perspective, keratinase activity by some has been reported to occur between neutral and alkaline pH condition. A good example would be *B. pumilus* GRK, which actively produced keratinase between pH 8.0 and 12.0, achieving the maximum keratinase activity at optimum initial pH 10.0 [18]. *Bacillus licheniformis* ER-15 similarly produced keratinase within the pH range of 7.0 to 10.0, and the optimum initial fermentation pH was 7.0 [14]. Reports abound on the alkalophilic nature of keratinase-producing *Bacillus* species. However, the acidophilic nature of *Bacillus* sp. FPF-1, which is the focus of the study, might be an embodiment of unique biotechnological potentials this bacterial strain might hold in the valorization value chain. 

**Figure 3:**
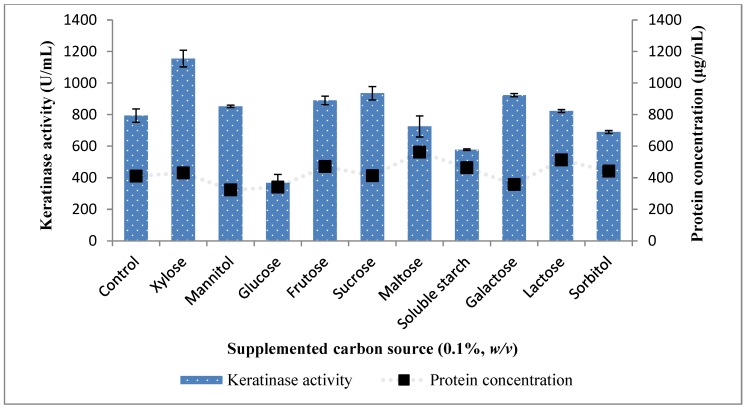
The influence of saccharide supplementation of medium for enhanced keratinase activity by *Bacillus* sp. FPF-1.

**Figure 4:**
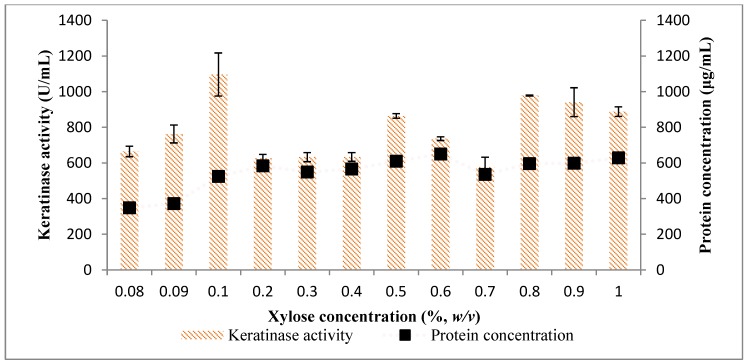
The effect of xylose concentrations on keratinase activity by *Bacillus* sp. FPF-1.

In consideration of the effect of carbon sources, other than the chicken feather, on the production of keratinases by *Bacillus* sp. FPF-1, xylose showed a considerable influence with keratinase yield at 1155.45 ± 52.71 (U/mL) (Figure 3). Subsequently, xylose concentration was varied from 0.08% to 1.0% (*w*/*v*), and 0.1% was the optimum concentration, as it yielded a keratinase concentration of 1096.36 ± 120.85 (U/mL) (Figure 4). Beyond 0.1%, the concentration of xylose in the medium the effect was not positive, as the keratinase concentration in the broth did not increase. The mechanism for which xylose in the fermentation medium influenced/enhanced keratinase activity is unclear; however, it may have upregulated the expression of keratinase gene in *Bacillus* sp. FPF-1. In a similar study, 0.5% xylose was used as the fermentation medium, and *Bacillus amyloliquefaciens* B6 achieved a higher concentration of keratinase [19].

Other carbon sources showed a different pattern of influence on keratinase activity by *Bacillus* sp. FPF-1. Glucose decreased keratinase activity, as the yield was at 367.27 ± 53.99 (U/mL) as compared to the control with yield of 793.64 ± 42.43 (U/mL). Perhaps it would be prudent to indicate that catabolite repression may have been responsible for the phenomenon observed, as it is a known factor with the utilization of simple sugars as against a complex substrate [20,21]. Keratinase activity with the addition of the fructose, sucrose, and galactose, respectively were 889.99 ± 26.99, 935.45 ± 42.43 and 923.63 ± 10.28 (U/mL). Keratinase yield with these carbon sources were slightly higher than the control (*P* > 0.05); hence, they may serve as good inducers for keratinase activity (Figure 3). Mannitol, maltose and lactose neither significantly influence keratinase activity nor repressed the production.

**Figure 5:**
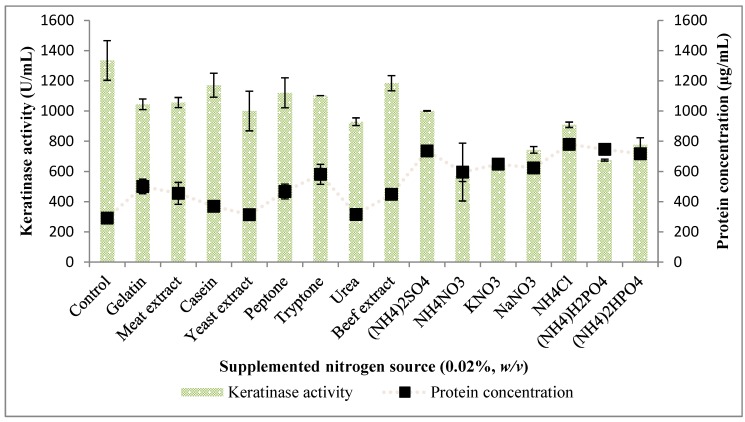
The effect of nitrogen supplementation on keratinase activity by *Bacillus* sp. FPF-1.

The supplementation of the fermentation medium with organic and inorganic nitrogen sources showed that *Bacillus* sp. FPF-1 produced lower quantities of keratinase across all the spectrum of supplemented nitrogen sources (Figure 5). *Bacillus* sp. FPF-1 would have preferentially utilized these nitrogen sources against the degradation of the keratinous biomass, and this concept may have accounted for the lower quantity of keratinases measured in the fermentation broth. 

Again, the total protein concentration in the fermentation broth was considerably higher with the ammonium salts; (NH_4_)_2_HPO_4_, (NH_4_)H_2_PO_4_, NH_4_Cl, NH_4_NO_3_ and (NH_4_)_2_SO_4_ yielded 717.42 ± 6.69, 746.57±1.11, 779.66 ± 25.63, 596.08 ± 191.6 and 736.33 ± 6.69 (µg/mL), respectively (Figure 5). It may be inferred that these ammonium salts influenced the production of some sorts of proteases, which are not keratinases. Consequently, the presence of more proteins in the fermentation supplemented with the salts compared to the control. These proteases may have acted upon cleaved units from the complex chicken feather structure. Complex nitrogen sources, including yeast extract, showed negligible effluence, which was statistically insignificant (*P* > 0.05). *Xanthomonas* sp. P5 and *B. licheniformis* MZK-3 were influenced to produce keratinases in the presence of additional nitrogenous source [22,23]. However, *Bacillus* sp. FPF-1 produced keratinases at a higher concentration in the absence of a nitrogen source other than chicken feathers, and an industrial prospect abound for the bacterial strain. 

**Figure 6:**
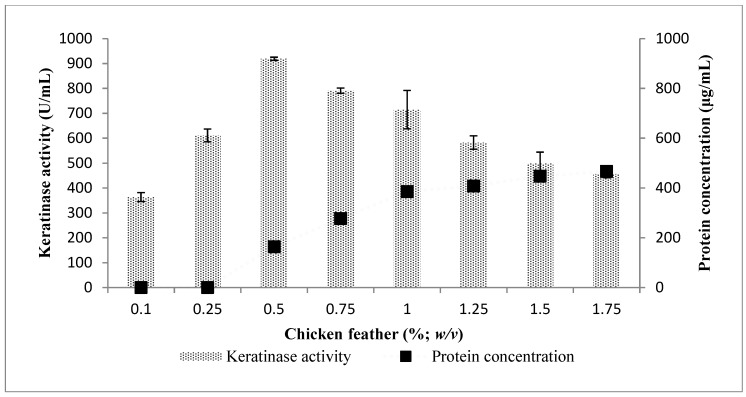
The effect of chicken feather concentration on keratinase activity by *Bacillus* sp. FPF-1.

The chicken feather concentration that was optimum for keratinase activity was at 0.5% (*w*/*v*) in the fermentation medium containing a 2% (*v*/*v*) starter culture with cell concentration of about 1 × 10^8^ CFU/mL (Figure 6). A further increase in the concentration of the chicken feather beyond 0.5% resulted in a constant decline in keratinase activity by *Bacillus* sp. FPF-1. However, the converse was the case with the total protein content measured in the fermentation broth; as the concentration of keratinase declined from 790.91 ± 10.29 to 714.55 ± 77.14 to 582.73 ± 26.99 (U/mL) with the respective chicken feather concentrations 0.75%, 1.0% and 1.25% (*w*/*v*), the total protein concentration in the fermentation medium increased as follows; 277.77 ± 2.23, 385.72 ± 10.03 and 408.57 ± 2.23 (µg/mL), respectively. 

The presence of a high concentration of total protein in the fermentation broth may be understood, as high potent keratinases breaking down the keratinous biomass faster than the producing bacteria is able to utilize, hence, the high concentration. Consequently, if the needed nitrogen source were available for assimilation, then naturally the organisms would downregulate the keratinase-expressing gene. Perhaps other enzymes that are different from keratinases are responsible for the cleavage of some peptides or proteins to forms assimilable by *Bacillus* sp. FPF-1. 

A similar phenomenon as described above has been reported previously. However, the reason advanced for the process was a reduction in dissolved oxygen concentration as a result of high viscosity of basal medium, hence, limiting the optimal activity of the microbial species [19,24]. Although the pattern of results was similar, we submit that a more logical explanation would lie on the simple concept of enzyme–substrate–gene regulation. 

**Figure 7:**
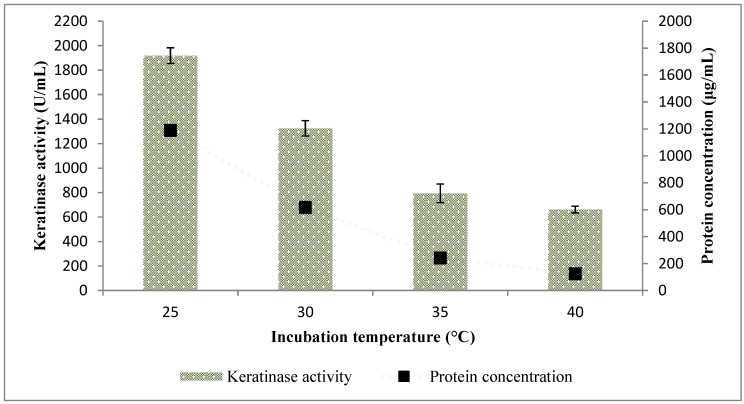
The effect of incubation temperature on keratinase activity by *Bacillus* sp. FPF-1.

The variation of the fermentation temperature showed that *Bacillus* sp. FPF-1 optimally produced keratinase at 25 °C (1918.18 ± 64.28 U/mL). As the fermentation temperature increased, keratinase activity decreased consistently (Figure 7). A similar pattern was observed with the total protein concentration of the fermentation broth; at 25 °C (1189.37 ± 23.39 µg/mL), 30 °C (617.36 ± 5.57 µg/mL), 35 °C (242.32 ± 16.71 µg/mL) and 40 °C (124.92 ± 2.29 µg/mL). The findings only buttress the supposition that keratinases have a high substrate-to-product conversion rate and that other enzymes, which may be heat labile, are also in play in the conversion of polymers to proteins.

However, organisms are unique and so are their characteristics; some keratinase-producing bacteria have functioned well at temperatures ranging from 25 to 40 °C [17,25,26]. Thermophilic keratinase-producing bacteria with optimum temperatures ranging from 50 to 70 °C have been reported [27,28,29]. However, *Bacillus* sp. FPF-1 may be graded as mesophilic, and the biotechnological significance would include a low energy input in a large-scale fermentation. 

**Figure 8:**
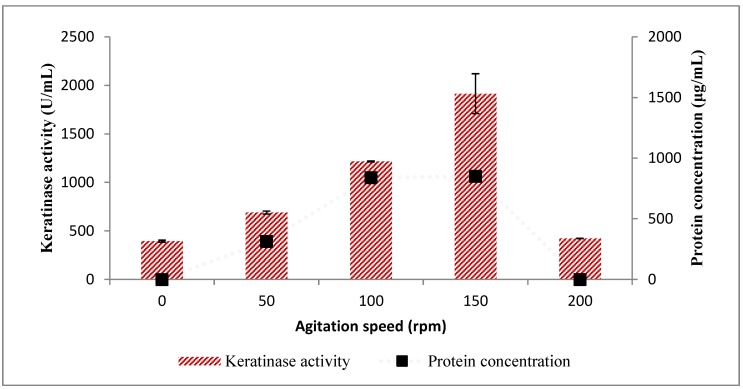
The effect of agitation speed on keratinase activity by *Bacillus* sp. FPF-1.

The impact of aeration on keratinase activity by *Bacillus* sp. FPF-1 showed that an agitation speed of 150 rpm was optimal (1914.54 ± 205.70 U/mL), and at higher agitation speed (200 rpm), keratinase activity drastically reduced (Figure 8). The total protein concentration followed a similar pattern as keratinase activity. Although agitation of a fermentation process suffices as a measure for aeration, it also serves other purposes, including homogeneity in nutrient access for cells in the fermentation process. 

The decline in keratinase activity may be attributed to the shear stress occasioned by the high agitation speed, which may have disrupted the integrity of the bacterial cells and, ultimately, affected the productivity [30]. However, if the integrity of the bacterial cells was not compromised, the conditions may not have been suitable for the desired metabolic processes. The peculiarity of organisms also influences their needs, and as such, *Pseudomona stutzeri* K4 [31], *Arthrobacter* sp. NFH5 [32] and *Bacillus altitudinis* GVC11 [33] were reported to optimally produce keratinases at an agitation speed peculiar to the respective organism.

### 2.4. Time Course Profile of the Keratinolytic Activity by Bacillus sp. FPF-1

**Figure 9:**
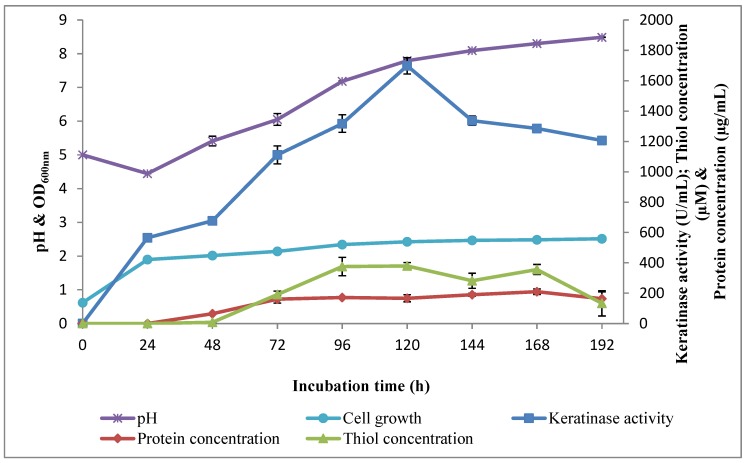
Time courses profile of keratinolytic activity by *Bacillus* sp. FPF-1 in an optimized fermentation medium at 25 °C and 150 rpm.

Keratinase activity time course by *Bacillus* sp. FPF-1 studied over 192 h showed that the active growth stage of the organism is very crucial, as keratinase concentration in the fermentation medium remained high; 1698.18 ± 53.99 (U/mL) was achieved in 120 h, and that was the optimum. Beyond 120 h, the keratinase concentration significantly declined. The pH of the fermentation broth decreased from the initial pH 5.0 to 4.5 ± 0.00 after 24 h of cultivation, and then it increased until 192 h of incubation, with a maximum value of 8.5 ± 0.01 (Figure 9). The plausible explanation for the observed trend would be the metabolites secreted by the microbe, as well the products of enzyme activities. The metabolites secreted at the early stages that made the medium more acidic may include the organic acid. At subsequent stages, alkaline-based metabolites would have dominated, and they would include nitrogen-related metabolites. Alkalization is brought about, predominantly, by the deamination of amino acids that emanate from keratin fragmentation [24]. 

The thiol concentration was negligible at 48 h of fermentation (Figure 9). Nevertheless, as fermentation progressed, thiol production gradually increased, and a maximum concentration of 379.53 ± 22.13 (μM) was observed after 96 h of fermentation. The thiol concentration declined after 168 h of fermentation, and it may be understood to reflect the reduction in keratinase activity occasioned by reduced disulfide bond breakage in the polymer. Moreover, the presence of thiol groups suggests effective disulfide bonds reduction and the liberation of free-sulfhydryl-group-containing products. The negligible presence of thiol groups during the early stages of fermentation period may be due to the utilization of the thiol groups’ containing products faster than the enzymes converted the products, or the thiol groups were not liberated. The concentration of thiol groups liberated by *Bacillus* sp. FPF-1 is significantly higher than has been reported for *Bacillus* sp. MBRL 575 [34] and *Stenotrophomonas maltophilia* R13 [25]. Consequently, *Bacillus* sp. FPF-1 presents a potential for exploitation as an industrially relevant microbe. 

### 2.5. Biodegradation of Intact Chicken Feather—Structural Evaluation

**Figure 10:**
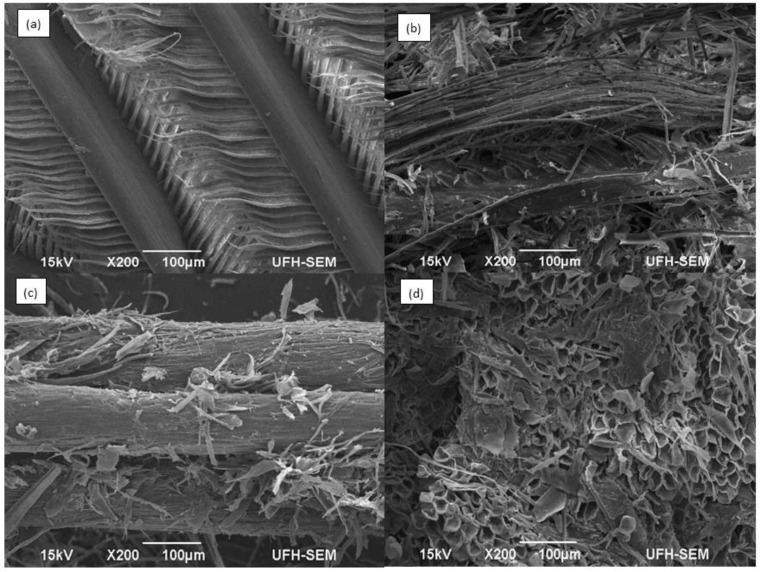
Electron micrography of feather degradation by *Bacillus* sp. FPF-1: (**a**) control (un-inoculated feather); (**b**) chicken feather at 24 h of fermentation; (**c**) feathers after 48 h fermentation; and (**d**) feathers after 72 h of cultivation.

The electron microscopic imaging of the chicken feather degradation showed the dismemberment of the structurally intact feather (Figure 10). As would be seen with Figure 10a, the feather is intact with all shafts and vane in place, and the integrity of the barbules were intact on the vane. However, as fermentation progressed, the barbules were completed degraded from the vane (Figure 10b), and the vane likewise was completely degraded in Figure 10c. All of these events occurred within 72 h of fermentation with *Bacillus* sp. FPF-1. In addition, beyond 72 h, it was clear that the organism had the potential to bio-convert the shaft, as a complete degradation was also achieved (Figure 10d). The imaging of chicken feather degradation by for *B. licheniformis* RG1 showed a similar pattern [35]. However, considerable chicken feather degradation was implemented by extensively studied *Bacillus licheniformis* PWD-1 after 10 days of incubation [36]. Again, the significance of the imaging process includes the understanding of the degradation pattern. Therefore, at a scale-up process, the nature of feedstock is essential for optimal product yields.

## 3. Materials and Methods

### 3.1. Keratin—Substrate Preparation

The keratin substrate was prepared from chicken feathers obtained from a local poultry processing farm. The feathers were thoroughly rinsed with distilled water and dried at 60 °C for 48 h. Dried feathers were milled with a pulverizer fitted with a 2 mm mesh and stored at room temperature, in an airtight container.

### 3.2. Sample Collection and Bacteria Isolation

Soil samples were collected from composting sites (32°43’48’S 27°1’32’E) at the Fort Cox College of Agriculture and Forestry, in the Raymond Mhlaba Local Municipality, Eastern Cape Province of South Africa. The samples were aseptically transported to the laboratory and processed within 6 h of collection. 

Basal salt media (BSM) was prepared as follows (g/L): K_2_HPO_4_, 0.3; KH_2_PO_4_, 0.4; MgCl_2_, 0.2; CaCl_2_, 0.22; NH_4_Cl, 0.5; powdered chicken feather (PCF), 10 [37]. About 1 g of the soil samples was inoculated in 99 mL of BSM and incubated at 30 °C, over a 5-day timeline, in an orbital shaker incubator (Labotec, South Africa), at 130 rpm. About 100 μL of the BSM-culture was spread on a PCF agar plate supplemented with 50 mg/L of nystatin. The plates were incubated at 30 °C for 48 h. 

### 3.3. Preliminary Assessment of Isolates for Proteolytic and Keratinolytic Activities 

The proteolytic activity of the isolates was evaluated by inoculating skimmed milk agar (SMA) plates containing (g/L); K_2_HPO_4_, 0.3; KH_2_PO_4_, 0.4; MgCl_2_, 0.2; CaCl_2_, 0.22; NH_4_Cl, 0.5; skimmed milk, 10 and bacteriological agar, 15 [9], with 10 μL of the standardized bacterial suspension (equivalent to 1 × 10^8^ CFU/mL). The inoculum was prepared and adjusted to 0.1 at 600 nm. The standardization approach was used for all inoculum preparation, unless stated otherwise. The plates were incubated at 30 °C for 24 h, and halo zone formation was an indication of casein hydrolysis. 

Isolates positive for proteolytic activity were then evaluated for keratinolytic activity. A 2% (*v*/*v*) standardized culture was inoculated into 98 mL of sterile basal media containing a 1% (*w*/*v*) intact chicken feather as sole carbon and nitrogen source [38]. The culture was incubated at 30 °C for 96 h in a rotary shaker (130 rpm). The flasks were monitored for observable feather degradation, and fermentation broth was analyzed accordingly. Stock culture of potent chicken feather degrading bacteria was maintained on PCF agar slants at 4 °C, for a fresh inoculum preparation, and another culture maintained in 20% glycerol at −86 °C for long-term storage.

### 3.4. Feather Hydrolysis Assay

The degree of feather degradation by the test bacteria was determined by the weight-loss approach, as previously described by Reddy et al. [18]. The fermentation broth was filtered (Whatman® no. 1, Johannesburg, Gauteng, South Africa) to recover undegraded feathers, and subsequently washed with distilled water to remove cell biomass. The residual feathers were oven-dried at 60 °C for 24 h, and the constant weight was achieved. The degree of feather degradation was calculated and expressed as a percentage, as shown in Equation (1) below:

(1) Percentage feather hydrolysis (%)=(1−RF/WF) × 100

where *RF* is the weight of residual feathers after fermentation; and *WF* is the weight of intact feathers before fermentation.

### 3.5. Identification of Keratinolytic Bacteria

The genomic DNA of the keratinolytic bacterial isolates was extracted by using the ZR Fungal/Bacterial DNA Kit^TM^ (Zymo Research, Irvine, CA, USA). The 16S target region was amplified, using the polymerase chain reaction (PCR), under standard conditions. The set of universal primers used for the 16S rRNA gene sequence amplification were 27f (5ʹ-AGAGTTTGATCMTGGCTCAG-3ʹ) and 1492r (5ʹ-CGGTTACCTTGTTACGACTT-3ʹ) as forward and reverse primers, respectively [39]. The amplicons were gel extracted (Zymo Research, Zymoclean^TM^ Gel DNA Recovery Kit, Irvine, CA, USA) and sequenced in forward and reverse directions, on the ABI PRISM^TM^ 3500xl Genetic Analyzer (Foster city, CA, USA). The sequence comparison with the reference sequences in the database was conducted by using the basic local alignment search tool (BLAST). The nucleotide sequences were submitted to the GenBank, with the following accession numbers: MG214993, MG214994, MG214995, MG214996 and MG214999.

### 3.6. Extracellular Keratinase and Total Protein Production

The extracellular keratinase and total protein was produced in a submerged fermentation with media constituents as (g/L): K_2_HPO_4_, 0.3; KH_2_PO_4_, 0.4; MgCl_2_, 0.2; CaCl_2_, 0.22; and CFP, 10. The fermentation flasks were inoculated with a 2% (*v*/*v*) starter culture (comparable to 1 × 10^8^ CFU/mL) and incubated at 30 °C for 120 h, in an orbital shaker (Labotec IncoShake (Pty) Ltd, Midrand, Gauteng, South Africa). After the triplicate submerged state fermentation, the culture broth was centrifuged at 15,000× *g* for 10 min, using micro-centrifuge (HERMLE Labortechnik GmbH, Wehingen, Germany) and the cell-free filtrate served as crude enzyme for analytical assays. 

### 3.7. Assay for Keratinase Activity and Total Protein Concentration

Keratinase activity assay was based on the method of Jaouadi et al. [40], with slight modification. The reaction mixture contained 0.5 mL of 10 g/L of keratin azure (Sigma-Aldrich, St. Louis, MO, USA) in 0.1 M Tris-HCl buffer, pH 7.5, and 0.5 mL of suitably diluted crude enzyme solution. The mixture was incubated at 37 °C for 1 h, with shaking at 220 rpm; after that, the reaction was stopped by placing the assay mixture in ice-cooled water for 10 min. The unutilized substrates were removed by centrifugation at 15,000× *g* for 10 min, and subsequently filtered (Millipore cellulose filters; 0.45 μm). The azo dye released in the filtrate was determined at 595 nm, using a SYNERGYMx 96 well microplate reader (BioTek Instrument Inc., Winooski, VT, USA). The control was treated at the same condition which contained the enzyme solution and buffer without the substrate. One keratinase unit was defined as the amount of enzyme causing an increase in absorbance of 0.01 per hour under the standard assay condition. 

The total protein concentration was estimated by using the Bradford method [41], with bovine serine albumin as a standard protein. The respective assays were done in triplicate, and the results presented were mean plus standard deviation.

### 3.8. Determination of Medium Thiol Concentration

The thiol concentration was determined in the filtrate, using the methods described by Ellman [42]. Briefly, 50 μL of 4 mg/mL of 5,5ʹ-dithiobis (2-nitrobenzoic acid) (DTNB) (Sigma-Aldrich, St. Louis, MO, USA) in 100 mM phosphate buffer, pH 8.0, was mixed with 500 μL of distilled water. Then, the cell-free filtrate (250 μL) was added in the mixture and allowed to stand at room temperature for 5 min, for stable color development. The absorbance of yellow-colored 2-nitro-5-thiobenzoic acid (TNB) that formed upon reduction of DTNB was measured at 412 nm. Un-inoculated broth treated in the same manner as above served as the control. Triplicate assays were carried out, and the results presented were mean plus standard deviation. 

### 3.9. Effect of Physicochemical Conditions on Keratinase Activity and Total Protein Production

The effect of physicochemical conditions on keratinase activity and total protein production was assessed by using the one-variable-at-a-time (OVAT) approach. The effect of pH was assessed through varying the initial fermentation pH from 4.0 to 10, at intervals of one unit. The incubation temperature was also varied from 25 to 40 °C, at intervals of 5 °C. The influence of the carbon sources, xylose, mannitol, glucose, fructose, sucrose, maltose, soluble starch, galactose, lactose and sorbitol, (Merck chemicals (Pty) Ltd, Modderfontein, Gauteng, South Africa) was likewise evaluated. Moreover, the concentrations of the carbon sources were initially at 0.1% (*w*/*v*) of the fermentation medium. The carbon sources with the optimum activity were further evaluated with concentration variation from 0.08% to 1% (*w*/*v*). The effects of the nitrogen sources, gelatin, yeast extract, malt extract, casein, peptone, tryptone, urea, beef extract, NH_4_SO_4_, NH_4_NO_3_, KNO_3_, NaNO_3_, NH_4_Cl, (NH_4_)H_2_PO_4_ and (NH_4_)_2_HPO_4_ (Merck chemicals (Pty) Ltd., Modderfontein, Gauteng, South Africa), were also investigated at a concentration of 0.02% (*w*/*v*). The effect of chicken feather concentration (0.1–1.75%, *w*/*v*) on keratinase activity and total protein production was also investigated. Lastly, the effect of agitation speed on keratinase activity and total protein production was determined at speeds ranging from 0 to 200 rpm, at intervals of 50 units. 

The optimum conditions were used to conduct the time course assay, and the formulated basal media contained the following (*w*/*v*): 0.03% K_2_HPO_4_, 0.04% KH_2_PO_4_, 0.02% MgCl_2_, 0.022% CaCl_2_, 0.1% xylose and 0.5% PCF. The flasks were inoculated with 2% (*v*/*v*) starter culture and incubated at 25 °C, for 192 h and 150 rpm. An aliquot of the fermentation broth was aseptically and periodically withdrawn to determine the keratinase activity, protein concentration, cell growth, thiol concentration and pH change. In each case, triplicate experiments were carried out, and the results presented were mean plus standard deviation.

### 3.10. Structural Studies: Scanning Electron Microscopy

A scan electron microscopy of the biodegraded feather was conducted in line with the described procedures of Gupta and Singh [43]. Briefly, degraded feather at different interval of fermentation was recovered, washed with distilled water and oven-dried at 60 °C, for 24 h. The residual feather was fixed on carbon-sided-tape mounted on the stub and coated with gold palladium for 5 min, using an ion coater (Eiko Engineering Co. Ltd, Hitachinaka, Ibaraki, Japan), and observed under scanning electron microscope (JEOL Ltd, Tokyo Japan). 

### 3.11. Statistical Analysis

Experiments were performed in triplicates. The data were subjected to analysis of variance, and the degree of freedom was set at *P* < 0.05 significance level. The analysis was conducted with the IBM Statistical Package for Social Science version 23 (Armonk, NY, USA).

## 4. Conclusions

Valorization of waste to valuable products is an integral part of the exploitation of renewable resources for sustainable development. The approach of value addition to wastes has fed extensively into the bio-economy of many nations. Thus, the potentials presented by *Bacillus* sp. FPF-1 as indicative of the excellent production of keratinases which bio-converts chicken feathers into proteins and related products. The process conditions showed an optimal keratinase yield at a weak acidic initial fermentation medium, a mesophilic temperature condition and moderate agitation speed. Spiking the fermentation medium with xylose enhanced the production of the enzyme of interest, and the opposite effect was achieved with the inclusion of additional nitrogen sources. The capacity to effectively utilize chicken feathers as a sole source of carbon and nitrogen by *Bacillus* sp. FPF-1 is indicative of the fact that a battery of enzymes is produced to effectively degrade the complex pertinacious polymer. The high concentration of total proteins in the fermentation broth is a strong indicator to the assertion. In addition, the potency of the keratinase was laid bare in the electron micrograph indicating the degradation pattern over time. *Bacillus* sp. FPF-1 presents a very good prospects vis-à-vis the unique enzymes, and enzyme products and the organism as a whole serving as an industrially relevant strain. Conclusively, the complete and effective degradation of chicken feathers by *Bacillus* sp. FPF-1 underpins its potential in the valorization of recalcitrant keratinous waste biomass from the agro sector into useful products.

## Figures and Tables

**Table 1:** Identification of keratinolytic bacterial isolates, using 16S rDNA sequence.

S/N	Isolate Code	Reference Sequence	Sequence Similarity (%)	Sequence Identity	NCBI Accession Number
1.	FPF-1	*Bacillus* cereus AB1 (MF800922) *Bacillus thuringiensis* WG20 (KY085971)	100	*Bacillus* sp. FPF-1	MG214993
2.	FPF-8	*Chryseobacterium culicis* R4-1A (NR_117008)	98	*Chryseobacterium* sp. FPF-8	MG214994
3.	FPF-10	*Brevibacillus parabrevis* C20 (KX832699)	99	*Brevibacillus* sp. Nnolim-K2	MG214995
4.	FPF-12	*Brevibacillus parabrevis* NAP3 (KJ872854)	99	*Brevibacillus* sp. FPF-12	MG214996
5.	FSS-1	*Brevibacillus* sp. BAB-6437 (KY672924)	99	*Brevibacillus* sp. FSS-1	MG214999

**Table 2:** Keratinolytic activities of the bacterial fermentation from chicken feathers.

S/N	Isolate	Keratinase Activity (U/mL)	Protein Concentration (μg/mL)	Thiol Concentration (μM)	Final pH	Feather Weight Loss (%)
1.	*Bacillus* sp. FPF-1	440.01 ± 20.57 ^c^	759.97 ± 22.29 ^d^	2206.59 ± 49.79 ^d^	8.09 ± 0.01 ^d^	86.0 ± 1.41 ^c^
2.	*Chryseobacterium* sp. FPF-8	260.0 ± 25.71 ^b^	279.35 ± 24.51 ^b^	1028.98 ± 88.53 ^c^	8.53 ± 0.02 ^e^	82.0 ± 1.41 ^c^
3.	*Brevibacillus* sp. Nnolim-K2	214.55 ± 5.14 ^a^	160.38 ± 38.99 ^a^	465.59 ± 11.07 ^a^	7.43 ± 0.02 ^b^	66.0 ± 2.83 ^ab^
4.	*Brevibacillus* sp. FPF-12	233.18 ± 12.21^ab^	287.23 ± 20.06 ^b^	461.69 ± 5.53 ^a^	7.35 ± 0.00 ^a^	62.5 ± 2.12 ^a^
5.	*Brevibacillus* sp. FSS-1	252.73 ± 5.14 ^ab^	639.42 ± 34.54 ^c^	755.11 ± 0.00 ^b^	7.51 ± 0.03 ^c^	69.5 ± 2.12 ^b^

The values are presented as the mean and standard deviation of triplicate experiments; the values without the same superscript letters down the column are significantly different (*P* < 0.05).
